# How are exclusively data journals indexed in major scholarly databases? An examination of four databases

**DOI:** 10.1038/s41597-023-02625-x

**Published:** 2023-10-25

**Authors:** Chenyue Jiao, Kai Li, Zhichao Fang

**Affiliations:** 1 School of Information Sciences, University of Illinois Urbana-Champaign, 501 E. Daniel St., Champaign, IL 61820 USA; 2 School of Information Sciences, University of Tennessee, Knoxville, 451 Communications Building 1345 Circle Park Drive, Knoxville, TN 37996 USA; 3 School of Information Resource Management, Renmin University of China, Beijing, 100872 China; 4 Centre for Science and Technology Studies (CWTS), Leiden University, Kolffpad 1, 2333 BN Leiden, the Netherlands

**Keywords:** Research data, Publishing

## Abstract

The data paper is becoming a popular way for researchers to publish their research data. The growing numbers of data papers and journals hosting them have made them an important data source for understanding how research data is published and reused. One barrier to this research agenda is a lack of knowledge as to how data journals and their publications are indexed in the scholarly databases used for quantitative analysis. To address this gap, this study examines how a list of 18 exclusively data journals (i.e., journals that primarily accept data papers) are indexed in four popular scholarly databases: the Web of Science, Scopus, Dimensions, and OpenAlex. We investigate how comprehensively these databases cover the selected data journals and, in particular, how they present the document type information of data papers. We find that the coverage of data papers, as well as their document type information, is highly inconsistent across databases, which creates major challenges for future efforts to study them quantitatively, which should be addressed in the future.

## Introduction

Research data has become one of the most important objects in the research system during the past decade. Researchers across knowledge domains are relying on larger quantities of data to understand their research topics, which has brought significant changes to how our research system works and how research is conducted^1,2^. In particular, it is commonly agreed that the increasing amount of research data has raised distinct new requirements for data collection, processing, publishing, and sharing^3^, which cannot be sufficiently fulfilled without support from new infrastructure^4^. One recent development in this area is the 2016 proposal of the FAIR principles as guidelines for various stakeholders in the e-Science domain to enhance the findability and usability of data objects^5^. The 15 principles form a clear and actionable framework for the development of data-related initiatives and have been embraced by many parties in the research community.

Another significant recent development concerning research data is the academic genre of the data paper, which gradually took shape in the early 2010s. It is officially defined as a “scholarly publication of a searchable metadata document describing a particular online accessible dataset, or a group of datasets, published in accordance to the standard academic practices”^6^. Serving as a descriptor and citable proxy of data objects in the bibliographic universe, it can make research data more findable, citable, and reusable under the current research infrastructure^7–9^, goals that are consistent with the FAIR principles^10,11^. Moreover, data papers are making it easier for research data to be peer-reviewed, a significant prerequisite for the integration of data objects into the research system^12,13^. From this perspective, we are also not trying to distinguish the variant names assigned to this type of documents, such as “data article” in *Data in Brief*, “data description paper” in *Earth System Science Data* and “data descriptor” by *Scientific Data*.

Over time, more journals have begun accepting data papers. In this research, all periodicals accepting data papers are termed *data journals*; and more specifically, we distinguish journals primarily publishing data papers (i.e., *exclusively data journals*; the operationalization of this concept is discussed in the Methods section) from the rest that accept data papers just as a genre in addition to research articles (i.e., *mixed data journals*), following how these categories are defined in previous studies^9,14^.

As data papers are becoming a popular way for researchers to publish their research data in many disciplines^14,15^, this new genre has become an important data source for investigating how data is used by scientists. This echoes increasing interest in research data from the field of quantitative science studies^16,17^. Numerous studies have been conducted using quantitative methods and large-scale datasets to understand the relationship between research data and scientific studies and outputs, such as how data objects are cited and/or mentioned in scientific publications^18–21^ and the disciplines behind the datasets^22^. The majority of existing research uses citations to data repositories, such as DataCite^23^ and the Inter-university Consortium for Political and Social Research (ICPSR) data repository^22,24^ as well as Clarivate’s Data Citation Index^25,26^, which is also primarily based on data repositories^27^. However, despite the growing importance of data papers, very few studies in this line of research have analyzed them directly, with a few exceptions based on small numbers of individual data journals^7,9,28^.

The absence of data papers from large-scale empirical studies represents a major gap in the existing research infrastructure for effectively tracing data papers. Efforts have been made to identify data journals^14,29^, but to our knowledge, no research has been conducted to understand how these journals and their publications are indexed in scholarly databases, such as the Web of Science (WoS) and Scopus, which are frequently used as the direct data source in quantitative science studies. This gap makes it harder for researchers to easily extract a large body of data papers from scholarly databases and analyze them, especially using quantitative methods.

To bridge this important gap, this research aims to examine the coverage of data journals and data papers published in these journals indexed in major scholarly databases used in quantitative science studies, including the WoS, Scopus, Dimensions, and OpenAlex. We selected a list of exclusively data journals from lists of data journals that have been compiled by other researchers. Using this list, we evaluated how data papers in these journals are indexed in the above databases, particularly from the perspectives of document types used to describe the publications and changes in coverage over time. More specific research questions include:

### RQ1: Which exclusively data journals are indexed in major scholarly databases?

Using various lists of data journals (discussed in Methods), we compile a list of exclusively data journals based on our operationalization of this concept and quantitatively examine their presence in the databases listed above. This will serve as the basis for future quantitative studies on the genre of data papers.

### RQ2: How are data papers in these journals indexed over time?

Building upon the survey of data journals in RQ1, we further examine how different databases index publications (most of which being data papers) from these exclusively data journals over time, to understand the coverage of this genre from a more granular and dynamic perspective.

### RQ3: Are data papers indexed accurately in terms of document type?

The last question aims to offer a survey of the extent to which data papers in the journals are labeled as data papers in the selected databases. Correct labeling is the first step for data papers to be distinguished from other types of publications (especially research articles) in a database and analyzed separately. Answers to this research question will lead to a better understanding of the gaps in the current infrastructure for data papers and facilitate more meaningful support of data publication in the future.

## Methods

### Identifying exclusively data journals

Data journals, as a new venue for data sharing and publishing, have gained increasing attention from scholars. There are many resources that provide lists of data journals. In this study, we resorted to the following resources to compile a list of exclusively data journals: (1) Candela and colleagues’ survey^14^, (2) an updated journal list by Walters^29^, (3) a list of data journals compiled by Kindling and Strecker^30^, (4) data journal lists created by academic libraries and other parties indexed by Google (e.g., the list of data journals created by the University of Pittsburgh available at: https://pitt.libguides.com/findingdata/datajournals), and (5) journals with “data” or “database” in the title included in the Journal Citation Reports or Scopus List of Journals.

From these sources, we further selected exclusively data journals based on the following criteria: (1) the journal primarily accepts data papers based on its statement of aims and scopes, operationalized as a greater than 50% share of data papers among all publications on the journal website, (2) the journal is active as of January 2023, and (3) the journal only publishes English-language articles. We manually examined all candidate journals against these criteria. For example, only about one-quarter of all publications in *Biodiversity Data Journal* are data papers, leading us to remove this periodical from the present study, despite the fact that it is mentioned as an important data journal in previous studies^6,7^. We also excluded *Arxius de Miscellania Zoologica*, which publishes data papers in Catalan, English, and Spanish. *Genomics Data* was excluded because it was published from 2013 to 2017; the journal is now part of *Data in Brief*. Finally, we selected the 18 journals shown in Table 1 as the analytical sample for this research.

**Table 1:** Complete list of data journals in our sample.

Data journal	Publisher	Initial year
Chemical Data Collections	Elsevier	2016
Data in Brief	Elsevier	2014
Earth System Science Data	Copernicus	2009
Freshwater Metadata Journal	Freshwater Information Platform	2014
Geoscience Data Journal	Wiley	2014
International Journal of Food Contamination	BMC	2014
IUCrData	International Union of Crystallography	2016
Journal of Chemical and Engineering Data	American Chemical Society	1959
Journal of Open Archaeology Data	Ubiquity Press	2012
Journal of Open Humanities Data	Ubiquity Press	2015
Journal of Open Psychology Data	Ubiquity Press	2013
Journal of Physical and Chemical Reference Data	AIP Publishing	1972
Nuclear Data Sheets	Elsevier	1971
Open Data Journal for Agricultural Research	sponsored by Wageningen University and Research Centre Library	2015
Open Health Data	Ubiquity Press	2013
Open Journal of Bioresources	Ubiquity Press	2014
Research Data Journal for the Humanities and Social Sciences	Brill	2016
Scientific Data	Nature	2014

### Collecting data papers from major databases

In this study, we strove to answer our research questions using the WoS, Scopus, Dimensions, and OpenAlex, as these are among the most commonly used large-scale bibliographic data sources in quantitative science studies.

For each database, we collected the metadata information of all publications from each journal in our final list that was indexed in the database. We used the online portal of the WoS and Scopus for data collection. As for Dimensions and OpenAlex, we retrieved the data from the in-house Dimensions database (version: June 2022) and OpenAlex database (version: October 2022) hosted at the Centre for Science and Technology Studies (CWTS) of Leiden University, respectively. We only considered papers published by the end of 2021.

### Comparing document types of our sample

The classification of document types varies by database. In Dimensions and OpenAlex, all publications from indexed data journals are classified as *Article*, whereas various document types appear in WoS and Scopus. Therefore, we only compared the document types between the latter two databases. Table 2 shows how the two main document types of interest, *Article* and *Data paper*, are defined by these two databases, as quoted from their documentation. Based on their definitions and their presentation in the data, we classified publications into these two types. Our classification also includes other document types, such as *Correction* and *Editorial material*, which are categorized as *Other* in this research. We note that, based on our examination, the WoS retrospectively assigned *Data paper* to articles published before 2016, when this type was introduced. However, it was unclear when the *Data paper* tag was introduced into Scopus. As a result, our analysis of the document type must be based on the data collected at this time.

**Table 2:** Document type policies in WoS and Scopus.

Database	Document type	Description
WoS (https://webofscience.help.clarivate.com/en-us/Content/document-types.html)	Article	Reports of research on new and original works that are considered citable. Includes research papers, brief communications, technical notes, chronologies, full papers, and case reports (presented like full papers) that were published in a journal and/or presented at a symposium or conference. Articles usually include author abstract, graphs, tables, and lists of cited references.
	Data paper	A scholarly publication describing a particular dataset or collection of datasets and usually published in the form of a peer-reviewed article in a scholarly journal. The main purpose of a data paper is to provide facts about the data (metadata, such as data collection, access, features etc.) rather than analysis and research in support of the data, as found in a conventional research article. A Data Paper will have a dual document type: Article; Data Paper. Prior to 2016, a Data Paper was processed as an Article only.
Scopus (https://www.elsevier.com/__data/assets/pdf_file/0007/69451/ScopusContentCoverageGuideWEB.pdf)	Article	Original research or opinion. Articles in peer-reviewed journals are usually several pages in length, most often subdivided into sections: abstract, introduction, materials & methods, results, conclusions, discussion, and references. However, case reports, technical and research notes and short communications are also considered to be articles and may be as short as one page in length. Articles in trade journals are typically shorter than in peer-reviewed journals, and may also be as brief as one page in length.
	Data paper	Searchable metadata documents describing an online accessible dataset, or group of datasets. The intent of a data paper is to offer descriptive information on the related dataset(s) focusing on data collection, distinguishing features, access, and potential reuse rather than information on data processing and analysis.

To examine the accuracy of document types in these databases, we collected the papers’ classification on the journal website and compared it with the document types assigned by the two databases. We focused on one journal in our list, *Scientific Data*, as a case study for two reasons: first, *Scientific Data* is the most influential data journal, especially in terms of impact factor; second, publications in this journal are searchable by article type on the website (https://www.nature.com/sdata/articles) so that it is easy to collect the classifications of each publication.

We collected 1,913 publications published by the end of 2021 in *Scientific Data* for this case study. The document types defined by the journal and the count of publications in each type are presented in Table 3. According to the definitions, we counted *Data descriptor* as *Data paper*, *Article* as *Article*, and all remaining categories as *Other* in our analysis. We then compared the count of publications in these categories from the journal website, WoS, and Scopus to examine the extent to which data journal publications are classified correctly in databases.

**Table 3:** Document types in *Scientific Data*.

Document type used by *Scientific Data*	Publications	Category definition (https://www.nature.com/sdata/publish/submission-guidelines#sec-1)
Data descriptor	1636	Detailed descriptions of research datasets, which focus on helping others reuse data, rather than testing hypotheses or presenting new interpretations.
Article	69	Reports on new policies, repositories, standards, ontologies, workflows, or any topic relating to the mechanics of data sharing.
Author correction	53	/
Analysis	44	A new analysis or meta-analysis of existing data, which highlights examples of data reuse or new findings.
Comment	41	Short commentaries or opinions on research data policy, workflows or infrastructure that don’t need to report a specific technology or finding.
Corrigendum	22	/
Publisher correction	17	/
Editorial	14	/
Erratum	10	/
Addendum	6	/
Retraction	1	/
Total	1913	/

## Results

### How are data journals and publications indexed?

The numbers of data journals and the year in which they were indexed in the database vary significantly among these four popular scholarly databases (see Table 4). Only eight data journals are indexed in WoS and 11 in Scopus, but Dimensions and OpenAlex have full coverage of the journal list. In terms of the indexed year, even though most of the journals are indexed in these databases in very similar manners, there are some notable differences, the majority of which are due to the fact that WoS is the most selective database among these four. Another notable observation is that the indexed years of *Journal of Chemical and Engineering Data* in three databases are prior to its established year. This is because a few publications were indexed by their first published dates instead of their formal published dates. Additionally, the fact that the WoS has later indexed years than most of the other databases is consistent with the fact that it has the most selective criteria for journals among the most popular databases^31^. It should also be noted that *IUCrData* and *Journal of Open Humanities Data* were indexed in Scopus from 2022, which is not covered by our publication window.

**Table 4:** Indexing of data journals in the four databases.

Data journal	Year established	First year of indexing			
		WoS	Scopus	Dimensions	OpenAlex
Chemical Data Collections	2016		2016	2016	2016
Data in Brief	2014	2018	2014	2014	2014
Earth System Science Data	2009	2012	2009	2009	2009
Freshwater Metadata Journal	2014			2014	2014
Geoscience Data Journal	2014	2014	2015	2012	2012
International Journal of Food Contamination	2014		2014	2014	2014
IUCrData	2016			2016	2016
Journal of Chemical and Engineering Data	1959	1965	1956	1956	1955
Journal of Open Archaeology Data	2012	2018		2012	2012
Journal of Open Humanities Data	2015			2015	2015
Journal of Open Psychology Data	2013			2013	2013
Journal of Physical and Chemical Reference Data	1972	1977	1972	1972	1972
Nuclear Data Sheets	1971	2003	1971	1971	1971
Open Data Journal for Agricultural Research	2015			2015	2015
Open Health Data	2013			2013	2014
Open Journal of Bioresources	2014		2019	2014	2014
Research Data Journal for the Humanities and Social Sciences	2016		2016	2016	2016
Scientific Data	2014	2014	2014	2014	2014

Table 5 presents the number of data journals established in three periods of time. We acknowledge that there may be other ways to classify the history of data journals; however, we selected the year 2014 because of its importance: multiple important data journals were established in this year, such as *Scientific Data* and *Data in Brief*. The earliest data journal is the *Journal of Chemical and Engineering Data* which was first published in 1959, followed by two others founded in the 1970s. We included these three data journals even though their inchoate publications may not be totally consistent with how data papers are defined today. A notable trend that can be observed from Table 5 is that most data journals in our list were established between 2014 and 2016, indicating that the data paper is a new and growing academic genre.

**Table 5:** Summary of the founding years of data journals.

Time period	Journals founded
Before 2000	3
2000–2013	4
2014–2016	11

Since the coverage and indexed year of data journals vary among databases, the number of publications also varies greatly. Table 6 illustrates the number of publications from each journal in the four databases. We found that OpenAlex has the most comprehensive coverage of publications, whereas the WoS has the fewest publications. For most journals, there is a small variance in the number of publications indexed in the databases, despite the identical publication window taken by these different databases. One notable issue we found in Table 6, compared to the data collected from *Scientific Data* (Table 3), is that three of the databases have more publications than the number of publications on the journal’s website per se. We double-checked our data collection pipeline and found that the extra publications are primarily ascribable to indexing errors, where the same publication is assigned different IDs and/or titles. To highlight this quality issue, we decided not to remove the duplicated publications from our analytical sample.

**Table 6:** Publications from each journal indexed in the databases.

Data journal	WoS	Scopus	Dimensions	OpenAlex
Chemical Data Collections	/	776	778	778
Data in Brief	5707	7567	7711	7823
Earth System Science Data	921	954	958	966
Freshwater Metadata Journal	/	/	51	52
Geoscience Data Journal	94	80	119	153
International Journal of Food Contamination	/	83	86	86
IUCrData	/	/	1536	1538
Journal of Chemical and Engineering Data	16910	18046	18088	18508
Journal of Open Archaeology Data	31	/	53	54
Journal of Open Humanities Data	/	/	51	51
Journal of Open Psychology Data	/	/	38	38
Journal of Physical and Chemical Reference Data	979	1039	1065	1090
Nuclear Data Sheets	802	1563	2607	2708
Open Data Journal for Agricultural Research	/	/	27	35
Open Health Data	/	/	26	22
Open Journal of Bioresources	/	27	50	50
Research Data Journal for the Humanities and Social Sciences	/	27	43	43
Scientific Data	1913	1943	1914	1943
Total	27357	32105	35201	35938

**Fig. 1:**
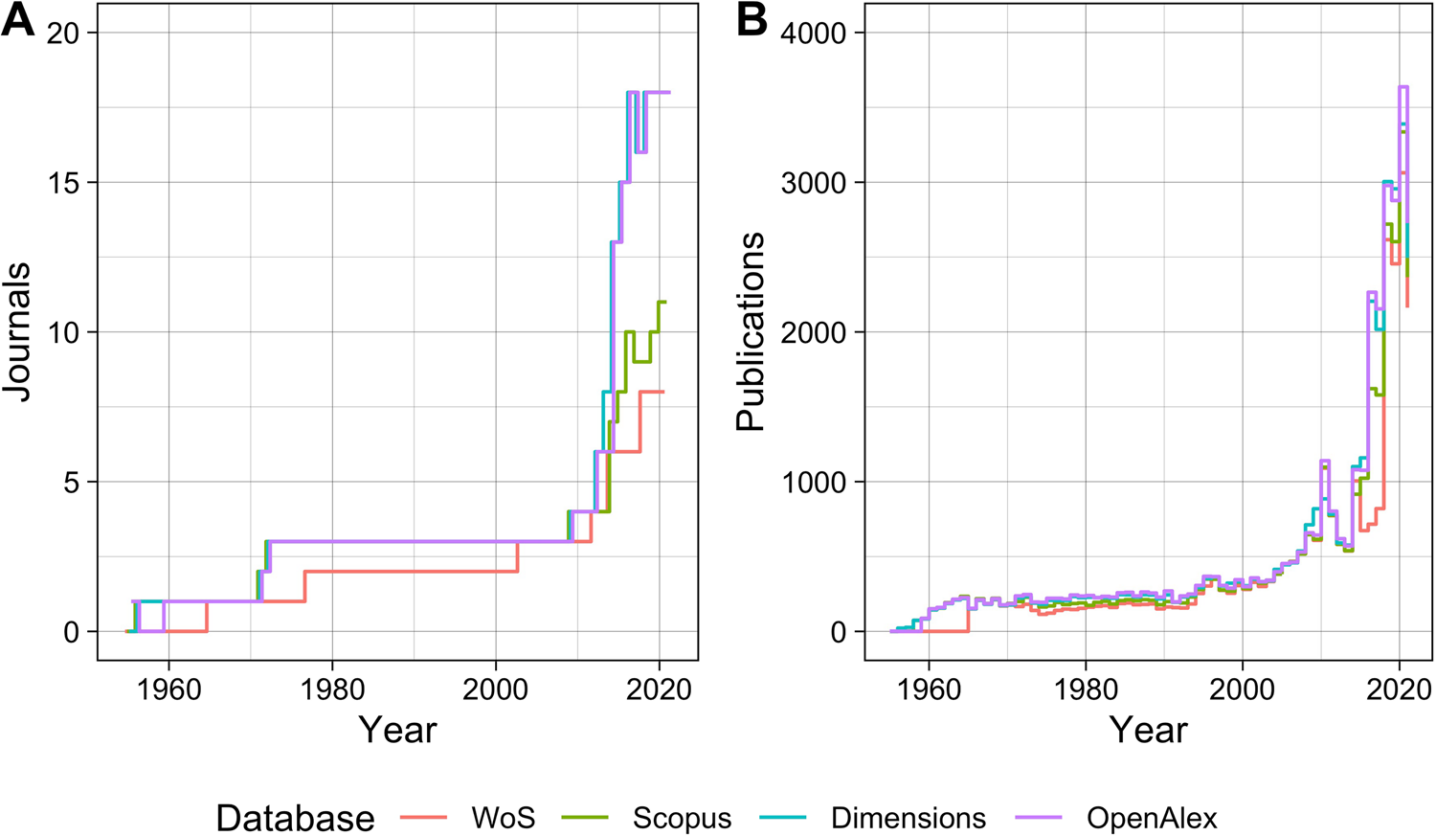
Numbers of data journals (Panel A) and data papers (Panel B) indexed in the four databases over time.

We further examined the numbers of journals and publications covered by each database over time. Figure 1 shows the trend on the journal level (Panel A) and the publication level (Panel B) respectively. We see a similar increasing trend for both journals and publications over time, especially from 2014 onwards. By the year of 2000, more than 15 exclusively data journals published more than 3,500 data papers every year, which shows the growth of this new academic genre. However, a notable difference between the databases can also be observed by comparing the two panels in Fig. 1: even though the number of journals covered by Dimensions and OpenAlex is much larger than that of the other two, their indexed publications are similar in size. This is because most of the journals covered by WoS and Scopus are larger than those omitted; being more selective, these two databases included journals that are potentially more established and important. As a result, despite the fairly large difference in the number of data journals from these databases, we can still use the WoS and Scopus to retrieve a large enough and potentially more representative sample of data papers from these exclusively data journals.

### Are publication types indexed consistently in the scholarly databases?

Each database has their own classification system of document types. Dimensions and OpenAlex assign *Article* to all data papers as they do for research articles, whereas WoS and Scopus have a specific category for data papers. Following the classification principles mentioned in Methods, Table 7 presents the share of all publications in each document type from the four databases. The distributions of publication in WoS and Scopus are similar to each other.

**Table 7:** Share of all data papers across the three document types.

Document type	Share in WoS	Share in Scopus	Share in Dimensions	Share in OpenAlex
Article	66.90%	66.34%	100%	100%
Data paper	26.84%	29.39%	/	/
Other	6.25%	4.27%	/	/

**Fig. 2:**
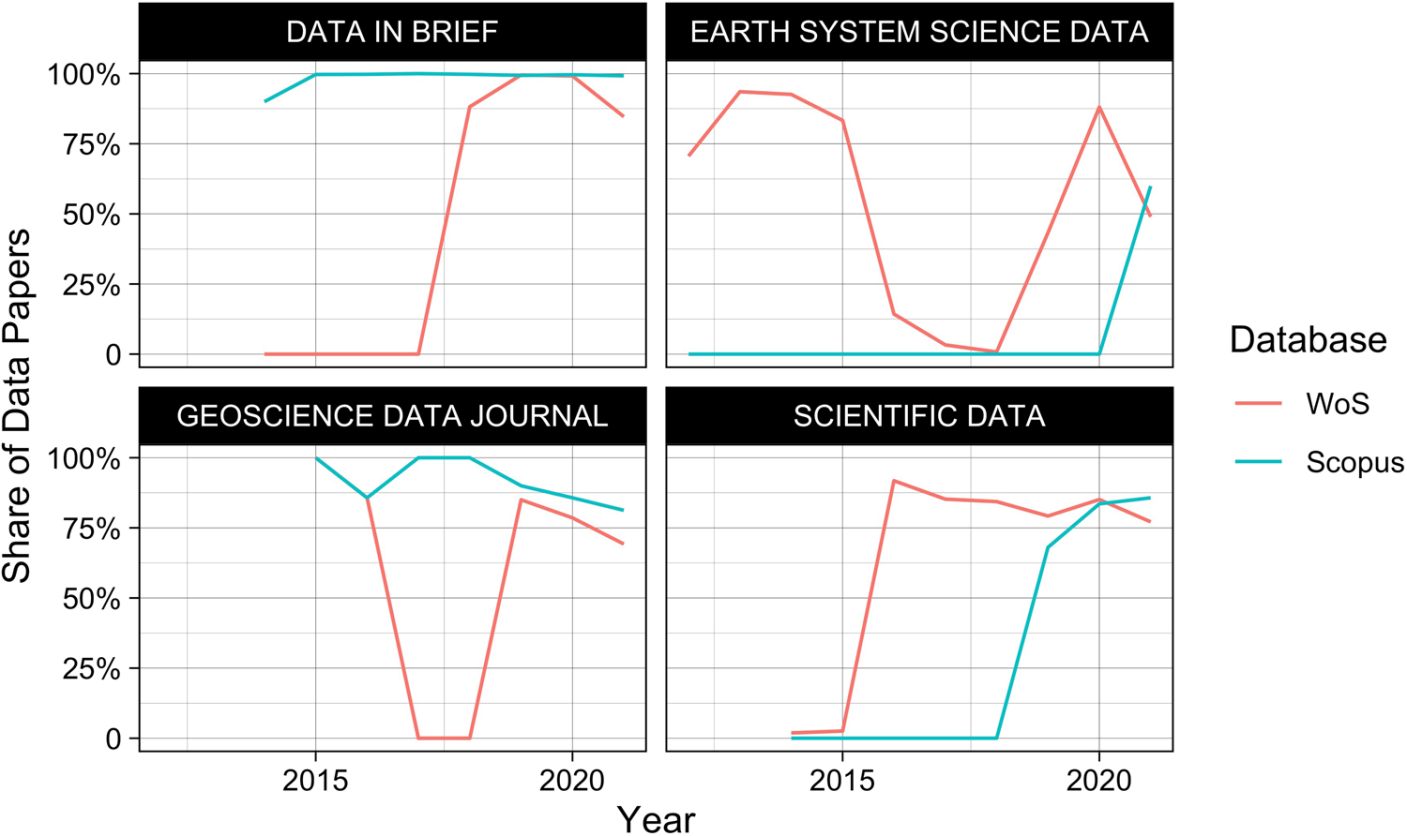
Share of data papers in each journal in each database over time.

We further evaluate the above trend from WoS and Scopus over time for four journals that are fully covered by both databases. Figure 2 shows that, despite the similar overall distributions in Table 7 between the two databases, there are vast differences in how document types are assigned in individual journals over time and between the two databases. This clearly shows that the assignment of the *Data paper* tag is far from consistent in any of these databases and cannot be reliably used to retrieve data papers in these two databases.

We further analyzed how publications from *Scientific Data* are indexed in WoS and Scopus, to understand the accuracy of document type assignment in a more granular manner. From the website of *Scientific Data*, there are 1,636 data papers, 69 articles, and 208 other publications, based on our classification. Table 8 shows how these publications are treated in the two databases. Even though both databases have many mislabeled articles, the WoS has a much higher accuracy (84.32%) than Scopus (59.27%).

**Table 8:** Correspondence between WoS and Scopus document types and those on the *Scientific Data* website.

		Document type from WoS		
		Data paper	Article	Other
**Document type from journal**	Data paper	1437	198	1
	Article	23	46	0
	Other	18	60	130
		**Document type from Scopus**		
		**Data paper**	**Article**	**Other**
**Document type from journal**	Data paper	903	719	14
	Article	0	69	0
	Other	0	45	162

## Discussion

In this work, we analyzed how exclusively data journals and publications in these journals are indexed in four major scholarly databases, as a first step towards establishing a comprehensive sample of data papers for future quantitative analyses. More specifically, we compiled a list of 18 exclusively data journals using existing efforts and analyzed how these journals and their publications are indexed and labelled by four such databases. Our results show significant inconsistencies in the indexing and labeling of data journals and papers by the popular databases, a major gap to be addressed by future efforts to improve the infrastructure that supports data publication and citation.

On the journal level, our results show that the two newer databases, Dimensions and OpenAlex, enjoy a strong advantage over the two more traditional databases, WoS and Scopus. The former two databases cover all of the exclusively data journals, whereas Scopus and WoS only cover 11 and 8 journals, respectively. Our results echo findings from past research that new databases in the market, such as Microsoft Academic Graph (the predecessor of OpenAlex) and Dimensions, are generally more comprehensive in terms of the research outputs indexed^32,33^. This trend is especially applicable to data journals because many of the exclusively data journals are relatively new and do not have many publications and citations, which makes these journals much less likely to be indexed in more established and selective databases.

Despite the large difference in the number of journals covered by these databases, we also find that the numbers of articles covered by the databases are much more similar to each other. Scopus and WoS cover about 90% and 75% of all articles in OpenAlex, respectively. This is because most of the data journals indexed in Scopus and WoS are also those with higher impact and more publications. This also shows that we will be able to collect a “good enough” and potentially more representative sample of data papers by simply using Scopus and, to a lesser extent, WoS.

Both of the above results suggest that the coverage of data publications in our existing knowledge infrastructure is still insufficient for such publications to be thoroughly and consistently retrieved and studied. Meta-research on data publication still needs to be based on scrupulous selection of the publication sample, given the limitations discussed above. In addition, we argue that this gap is also part of the insufficient integration of research data into the research system and can have strong negative impacts on how research data will be reused. Even though most of the data journals require the authors to supply the link to the data repository page in the data paper^14^, however, as a published document, such data papers can still be cited alone to represent a dataset. As a result, the data publication is an important method for research data to be identified in scholarly databases, which are fundamental data sources in various quantitative studies of science fields.

Beyond the data journals and papers indexed in the databases, we also examined the document type tag used for data papers in these databases, as this is the metadata element that will need to be used to retrieve data articles. Among the four databases we examined, all data papers are counted as regular research articles in Dimensions and OpenAlex, making it very challenging for researchers to acquire a full sample of data papers from them, despite their more comprehensive coverage of data papers. This is consistent with existing empirical evidence that the document type tag suffers from quality issues in most scholarly databases^34,35^ but the metadata quality in these emerging databases is often lower than in the more established databases^33,36^. By comparison, in Scopus and WoS, even though the *Data paper* document type is defined and used, papers bearing this label were introduced into these databases in different years, which contributes to inconsistent encoding of data papers. More importantly, we also find a stark gap in how publications in some of the data journals are encoded in these two databases. Through a more granular analysis using the case of *Scientific Data*, we find that the accuracy of this metadata element is significantly higher in WoS than in Scopus, which compensates for the less comprehensive coverage of data papers in the former data source.

Based on the results above, we argue that the inconsistent policies and implementation of the *Data paper* document type between popular scholarly databases pose a major issue for a more comprehensive identification of data papers from academic journals and the understanding of their roles in the research system. This is especially so given the facts that (1) the document type is the most important marker to distinguish data papers from research papers in scholarly databases and (2) many data papers are published in mixed data journals, where data papers and research articles are published together. As a result, the inaccuracies of this label in scholarly databases strongly prevents a thorough sample of data papers from being established for future quantitative studies. As a result, we believe this is an important issue to be solved through more communications among database vendors, data journal publishers and the open science community, so that research data will have great visibility in the research infrastructure and the quality of scholarly databases will be improved.

To sum up, our results highlight major limitations in existing scholarly databases to index and label data papers, an emerging and important representation of research data across various knowledge domains. This will lead to future efforts to improve our research infrastructure to support data-driven research and establish a more comprehensive and representative sample of data journals and publication for future metadata-analyses.

However, as the first step towards achieving the goals, our research has a major limitation that we are only focusing on exclusively data journals in this research, which offers only a partial view of the landscape of data publication. Whereas these journals are most strongly connected to data publication, many data papers are also published in mixed data journals, along with regular research articles. As the next step of the project, we will focus on identifying data papers from such mixed journals and understanding how data papers are published in these journals. In particular, we will design and experiment machine learning algorithm to distinguish data papers from research articles in mixed data journals.

Moreover, we will combine exclusively and mixed data journals to construct a large dataset of data papers, which will be critical for establishing a more comprehensive understanding of data publication. By using this novel dataset, we will be able to investigate new questions that are critical to the understanding of the relationship between research data and knowledge. Such questions include how the publishing and reusing of research data is connected to the discipline, gender, geography, and institution of researchers. These perspectives are central to the overall science studies communities and the investigation of how research data is produced and consumed from these perspectives will contribute to a better integration of data with the research system and have strong implications for future data-related research policies^37,38^.

## Data Availability

The raw data examined in this research is available at Figshare repository^39^.

## Code availability

The code used in this research is available at Figshare repository^39^.

## References

[1] Hey, T., Tansley, S., Tolle, K. M. & others. *The fourth paradigm: data-intensive scientific discovery*. vol. 1 (Microsoft research Redmond, WA, 2009).

[2] Borgman CL (2012). The conundrum of sharing research data. J. Am. Soc. Inf. Sci. Technol..

[3] Chen J (2013). Big data challenge: a data management perspective. Front. Comput. Sci..

[4] Chawinga WD, Zinn S (2019). Global perspectives of research data sharing: A systematic literature review. Libr. Inf. Sci. Res..

[5] Wilkinson, M. D. *et al*. The FAIR Guiding Principles for scientific data management and stewardship. *Sci. Data* **3** (2016). 10.1038/sdata.2016.18 PMC4792175 26978244

[6] Chavan V, Penev L (2011). The data paper: a mechanism to incentivize data publishing in biodiversity science. BMC Bioinformatics.

[7] Li K, Greenberg J, Dunic J (2020). Data objects and documenting scientific processes: An analysis of data events in biodiversity data papers. J. Assoc. Inf. Sci. Technol..

[8] Gorgolewski K, Margulies DS, Milham MP (2013). Making data sharing count: a publication-based solution. Front. Neurosci..

[9] Li, K. & Jiao, C. The data paper as a sociolinguistic epistemic object: A content analysis on the rhetorical moves used in data paper abstracts. *J. Assoc. Inf. Sci. Technol*. 1–13, 10.1002/asi.24585 (2021).

[10] Schöpfel J, Farace D, Prost H, Zane A (2020). Data papers as a new form of knowledge organization in the field of research data. KO Knowl. Organ..

[11] Groth P, Cousijn H, Clark T, Goble C (2020). FAIR data reuse–the path through data citation. Data Intell..

[12] Costello MJ, Michener WK, Gahegan M, Zhang Z-Q, Bourne PE (2013). Biodiversity data should be published, cited, and peer reviewed. Trends Ecol. Evol..

[13] Mayernik MS, Callaghan S, Leigh R, Tedds J, Worley S (2015). Peer review of datasets: When, why, and how. Bull. Am. Meteorol. Soc..

[14] Candela L, Castelli D, Manghi P, Tani A (2015). Data journals: A survey. J. Assoc. Inf. Sci. Technol..

[15] Griffiths A (2009). The Publication of Research Data: Researcher Attitudes and Behaviour. Int. J. Digit. Curation.

[16] Silvello G (2018). Theory and practice of data citation. J. Assoc. Inf. Sci. Technol..

[17] Cousijn, H., Feeney, P., Lowenberg, D., Presani, E. & Simons, N. Bringing citations and usage metrics together to make data count. *Data Sci. J*. **18** (2019).

[18] Zhao M, Yan E, Li K (2018). Data set mentions and citations: A content analysis of full-text publications. J. Assoc. Inf. Sci. Technol..

[19] Färber, M., Albers, A. & Schüber, F. Identifying Used Methods and Datasets in Scientific Publications. in *SDU@ AAAI* (2021).

[20] Lafia, S. *et al*. Detecting Informal Data References in Academic Literature. (2021).

[21] Gregory, K. *et al*. Tracing data: A survey investigating disciplinary differences in data citation. *Quant. Sci. Stud*. 1–51, 10.1162/qss_a_00264 (2023).

[22] Fan W, Jeng W, Tang M (2023). Using data citation to define a knowledge domain: A case study of the Add‐Health dataset. J. Assoc. Inf. Sci. Technol..

[23] Robinson-Garcia N, Mongeon P, Jeng W, Costas R (2017). DataCite as a novel bibliometric source: Coverage, strengths and limitations. J. Informetr..

[24] Lafia S, Fan L, Thomer A, Hemphill L (2022). Subdivisions and crossroads: Identifying hidden community structures in a data archive’s citation network. Quant. Sci. Stud..

[25] Robinson-García N, Jiménez-Contreras E, Torres-Salinas D (2016). Analyzing data citation practices using the data citation index. J. Assoc. Inf. Sci. Technol..

[26] Park H, Wolfram D (2019). Research software citation in the Data Citation Index: Current practices and implications for research software sharing and reuse. J. Informetr..

[27] Force MM, Robinson NJ (2014). Encouraging data citation and discovery with the Data Citation Index. J. Comput. Aided. Mol. Des..

[28] McGillivray B (2022). Deep Impact: A Study on the Impact of Data Papers and Datasets in the Humanities and Social Sciences. Publications.

[29] Walters, W. H. Data journals: incentivizing data access and documentation within the scholarly communication system. *Insights* **33** (2020).

[30] Kindling M, Strecker D (2022). Zenodo.

[31] Norris M, Oppenheim C (2007). Comparing alternatives to the Web of Science for coverage of the social sciences’ literature. J. Informetr..

[32] Singh VK, Singh P, Karmakar M, Leta J, Mayr P (2021). The journal coverage of Web of Science, Scopus and Dimensions: A comparative analysis. Scientometrics.

[33] Visser M, Van Eck NJ, Waltman L (2021). Large-scale comparison of bibliographic data sources: Scopus, Web of Science, Dimensions, Crossref, and Microsoft Academic. Quant. Sci. Stud..

[34] Donner P (2017). Document type assignment accuracy in the journal citation index data of Web of Science. Scientometrics.

[35] Yeung AWK (2019). Comparison between Scopus, Web of Science, PubMed and publishers for mislabelled review papers. Curr. Sci..

[36] Meho LI, Yang K (2007). Impact of data sources on citation counts and rankings of LIS faculty: Web of Science versus Scopus and Google Scholar. J. Am. Soc. Inf. Sci. Technol..

[37] Larivière V, Ni C, Gingras Y, Cronin B, Sugimoto CR (2013). Bibliometrics: Global gender disparities in science. Nature.

[38] Sugimoto CR, Weingart S (2015). The kaleidoscope of disciplinarity. J. Doc..

[39] Jiao C, Li K (2023). Figshare.

